# Oral Glucose Tolerance Test (OGTT) Evidence for the Postprandial Anti-Hyperglycemic Property of *Salacca zalacca* (Gaertn.) Voss Seed Extract

**DOI:** 10.3390/molecules28196775

**Published:** 2023-09-23

**Authors:** Vilasinee Hirunpanich Sato, Savita Chewchinda, Arman Syah Goli, Hitoshi Sato, Jannarin Nontakham, Boonyadist Vongsak

**Affiliations:** 1 Department of Pharmacology, Faculty of Pharmacy, Mahidol University, Bangkok 10400, Thailand; vilasinee.sat@mahidol.ac.th (V.H.S.); syahgoliarman@gmail.com (A.S.G.); 2 Department of Food Chemistry, Faculty of Pharmacy, Mahidol University, Bangkok 10400, Thailand; savita.che@mahidol.ac.th; 3 Division of Pharmacokinetics and Pharmacodynamics, Department of Pharmacology, Toxicology and Therapeutics, School of Pharmacy, Showa University, Tokyo 142-855, Japan; hitoshi.sato.showa@gmail.com; 4 Clinical Research Section, Division of Research and Academic Support, National Cancer Institute, Bangkok 10400, Thailand; jannarinn@gmail.com; 5 Pharmaceutical Innovations of Natural Products Unit (PhInNat), Burapha University, Chonburi 20131, Thailand; 6 Faculty of Pharmaceutical Sciences, Burapha University, Chonburi 20131, Thailand

**Keywords:** alpha-glucosidase, glucose uptake, diabetes, *Salacca*, salak seed extract, oral glucose tolerance test, postprandial hyperglycemia

## Abstract

Salak seed extract (*Salacca zalacca*) is known for its high antioxidant content and low caffeine levels, making it a promising candidate for the development of value-added health products. However, there is a lack of scientific evidence for its anti-hyperglycemic effects. To address this, we investigated the in vitro and in vivo anti-hyperglycemic and antioxidant effects of salak seed extract. The HPLC chromatogram of salak seed extract shows a prominent peak that corresponds to chlorogenic acid. In vitro studies revealed that salak seeds inhibited α-glucosidase activity and glucose uptake in Caco-2 cells in a concentration-dependent manner, while also exhibiting antioxidant properties. The extract exhibits a non-competitive inhibition on α-glucosidase activity, with an IC_50_ and K_i_ of 16.28 ± 7.22 and 24.81 μg/mL, respectively. In vivo studies utilizing streptozotocin-nicotinamide-induced diabetic mice showed that the extract significantly reduced fasting blood glucose (FBG) levels in the oral glucose tolerance test. Continuous administration of the salak seed extract resulted in lower FBG levels by 13.8% as compared with untreated diabetic mice, although this change was not statistically significant. The estimated LD_50_ value of salak seed extract exceeds 2000 mg/kg, and no toxicity symptoms have been detected. Our research supports that salak seed extract has the potential to serve as a functional food or supplement that may be beneficial in reducing postprandial hyperglycemia among people with type 2 diabetes. This effect was explained by the salak’s inhibitory mechanisms of glucose absorption due to inhibition of both α-glucosidase activity and intestinal glucose uptake, coupled with its antioxidant effects.

## 1. Introduction

Salak (*Salacca zalacca* (Gaertn.) Voss) is native to Indonesia, Malaysia, and other Southeast Asian countries, including Thailand, where it is known locally as snake fruit [1]. It is a fruit-producing species of palm tree (family Arecaceae) with approximately 30 different cultivars. These are categorized based on fruit size, pericarp color, taste, and texture [2]. Phytochemical and nutritional analyses of ripe snake fruit have detected numerous substances beneficial to humans, including phenolics, flavonoids, minerals, and vitamins. Mass spectrometric analysis has identified the presence of chlorogenic acid, catechin, (−)-epicatechin, and various proanthocyanidins [3,4]. These phenolic acids extracted from the fruit and peel parts of salak have been extensively researched for their antioxidant, anti-inflammatory, anti-mutagenic, and anti-hyperglycemic properties [5,6,7,8]. When extracted with 60% and 100% ethanol, the flesh of salak fruit exhibited inhibitory effects on α-glucosidase, with 50% inhibitory concentrations (IC_50_) of 16 μg/mL and 20 μg/mL, respectively [9]. After salak fruit was extracted with 60% ethanol, a previous study investigated the administration of the extract to obese diabetic rats and found a significant reduction in blood glucose and normalization of blood lipid profile [10]. Additional evidence for the therapeutic potential of salak fruit in the management of hyperglycemia has been provided by experiments with streptozotocin (STZ)-induced diabetic rats, in which salak fruit vinegar was found to reduce blood sugar [11].

The seed kernels of the salak fruit are reported to contain chlorogenic acid, linoleic acid, triacylglycerols, and β-sitosteryl-3β-glucopyranoside-6′-*O*-fatty acid esters. Among these compounds, chlorogenic acid is the most commonly observed phenolic acid in salak seeds [12], comprising a group of phenolic esters formed from caffeic and ferulic acids. It is present in numerous plants, including coffee, fruits, and vegetables. Recently, chlorogenic acid has been found to exert various pharmacological effects, including antioxidant, neuroprotective, and antidiabetic properties [6,7,13].

Salak seeds are rich in nutrients such as fat (0.48%), protein (4.22%), and carbohydrates (38.9%), while also containing chlorogenic acid as an active compound [13]. Although not commonly consumed as food, salak seeds have shown promise as a potential source of income through the development of various health products. For example, coffee with low levels of caffeine but high levels of antioxidants can be derived from salak seeds. This could be promoted as a promising natural, low-caffeine alternative for coffee enthusiasts [12,13,14].

Despite the efforts to develop functional health products from salak seed waste, a limited number of studies have been conducted on the pharmacological activities of salak seeds, particularly focusing on their antidiabetic potential. The current study aimed to elucidate the antidiabetic activities of salak seed extract and the mechanisms underlying its antihyperglycemic effects, both in vitro and in vivo, utilizing an STZ-nicotinamide (NA)-induced type 2 diabetes mellitus (STZ-NA induced diabetic) mouse model. Furthermore, the current study investigated the antioxidant and lipid peroxidation effects of salak seeds.

## 2. Results

### 2.1. Quantitative Phytochemical Analysis by High-Performance Liquid Chromatography (HPLC)

**Figure 1:**
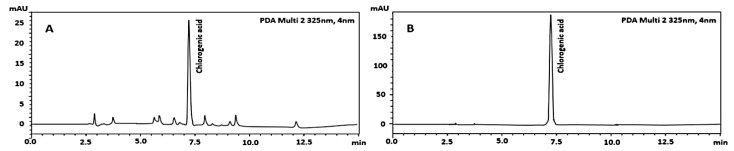
HPLC chromatograms of salak seed extract (**A**) and chlorogenic acid (**B**).

The HPLC chromatogram of salak seed extract showed a prominent peak at a retention time of 7.2 min, which corresponds to the chlorogenic acid reference standard (Figure 1). The chlorogenic acid content in the extract was calculated to be 0.55 ± 0.04% *w*/*w*.

### 2.2. Antioxidant Effects of Salak Seed Extract In Vitro

**Figure 2:**
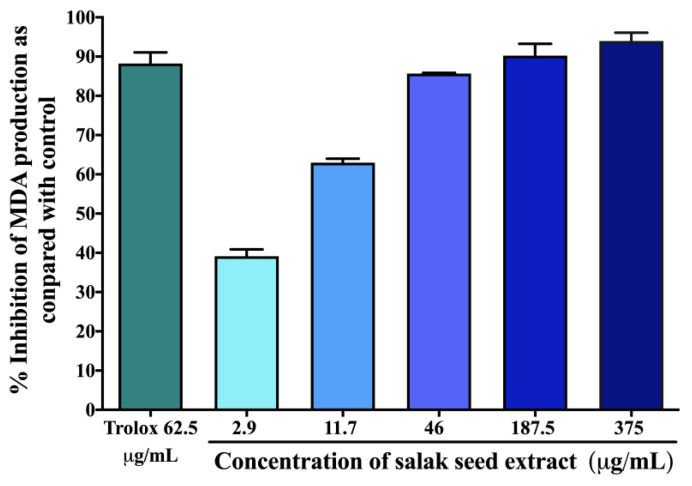
Effects of the salak seed extract on MDA production using thiobarbituric acid reactive substances (TBARS) assay. Data are expressed as mean ± SD (n = 3).

Table 1 summarizes the total phenolic content, ferric-reducing antioxidant power (FRAP) value, and 50% inhibitory concentration (IC_50_) values for 2,2-diphenyl-1-picrylhydrazyl (DPPH) and 2,2’-azinobis (3-ethylbenzothiazoline-6-sulfonic acid (ABTS) assays in the salak seed extract, in comparison with those of ascorbic acid used as a positive control. The measured extract exhibited a concentration-dependent inhibitory effect on the generation of malondialdehyde (MDA), as depicted in Figure 2. The IC_50_ value of the extract was determined to be 4.34 ± 1.71 µg/mL. In comparison, the positive control, Trolox, revealed an IC_50_ value of 0.011 ± 0.005 µg/mL.

### 2.3. Hypoglycemic Effects of Salak Seed Extract In Vitro

#### 2.3.1. Glucose Uptake in Caco-2 Cells

**Figure 3:**
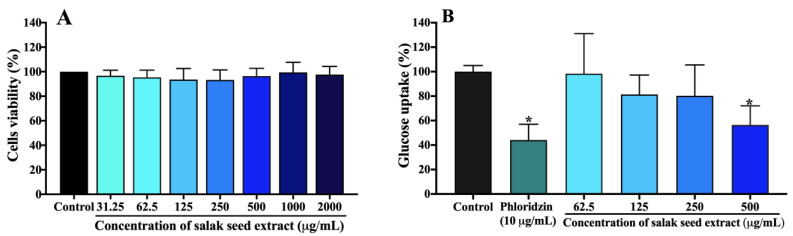
Effect of the salak seed extract on cell viability (**A**) and glucose uptake (**B**) in Caco-2 cells. Data are expressed as mean ± SD (n = 3). * *p* < 0.05 vs. control.

As shown in Figure 3A, salak seed extract at the concentration range of 31.25–2000 µg/mL did not affect the viability of Caco-2 cells when compared to a control, indicating no toxic effect on the cells. Consequently, a concentration range of 62.5–500 µg/mL of salak seed extract was chosen to conduct further studies aimed to assess glucose absorption in Caco-2 cells.

Figure 3B shows the average glucose uptake (%) in Caco-2 cells treated with salak seed extract compared to the untreated control. Phloridzin used as a positive control produced a significant decrease in glucose uptake of approximately 44% compared to the control (*p* < 0.05). The salak seed extract concentration-dependently inhibited glucose uptake in Caco-2 cells compared to the control, with an IC_50_ of 0.54 ± 0.13 mg/mL.

#### 2.3.2. α-Glucosidase Activity

**Figure 4:**
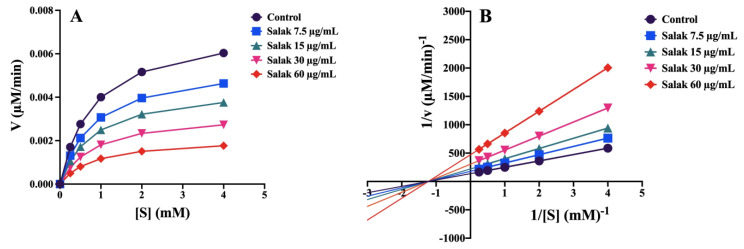
Michaelis-Menten (**A**) and Lineweaver-Burk plot (**B**) for the kinetic analysis of the α-glucosidase inhibitory activity of the salak seed extract. 1/[S] and 1/v are the reciprocals of substrate concentration (mM)^−1^ and reaction velocity (µM/min)^−1^, respectively.

Salak seed extract exhibited a concentration-dependent inhibition of α-glucosidase activity, which was higher than that exerted by acarbose (the positive control). The IC_50_ values of salak seed extract and acarbose were 16.28 ± 7.22 µg/mL and 370.99 ± 12.43 μg/mL, respectively. Figure 4A shows the Michaelis–Menten analysis of the inhibition induced by salak seed extract. An enzyme kinetic analysis was performed using Lineweaver–Burk plots (Figure 4B), demonstrating that salak seed extract inhibited α-glucosidase activity in a non-competitive manner (Figure 4B). The kinetic parameters, V_max_, K_m_, and K_i_, were calculated to be 0.0073 μM/min, 0.82 mM, and 24.81 μg/mL, respectively.

### 2.4. In Vivo Antihyperglycemic Activity of Salak Seed Extract

#### 2.4.1. Oral Acute Toxicity Test

Oral administration of salak seed extract at a dose of 2000 mg/kg did not result in any observable symptoms of toxicity or behavioral changes when compared to the control group (Table 2). Furthermore, no mouse mortality was observed during the 14-day observation period (Table 3). The body weight of all mice remained relatively stable throughout the two weeks of observation, and there was no significant difference in body weight between the control group and the group treated with salak seed extract. According to the OECD toxicity test guideline 425, this indicates that the lethal dose (LD_50_) value of salak seed extract exceeds 2000 mg/kg.

#### 2.4.2. Oral Glucose Tolerance Tests (OGTT) of Normal and Diabetic Mice

**Figure 5:**
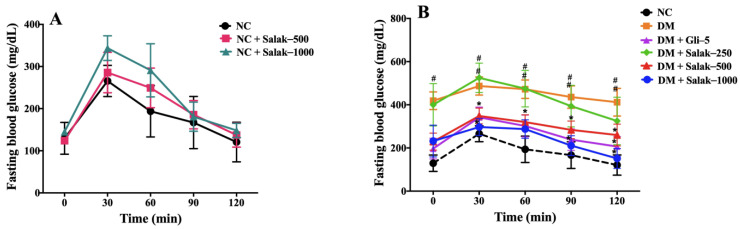

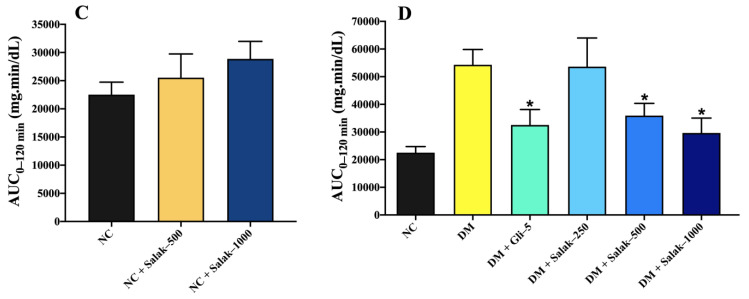
Effects of the salak seed extract on FBG in normal mice (**A**) and DM mice (**B**) and area under the curve (AUC_0–120 min_) of normal mice (**C**) and DM mice (**D**) in oral glucose tolerance test. Data are expressed as mean ± SEM (n = 6–8/group). # *p* < 0.05 vs. NC, * *p* < 0.05 vs. DM. NC: Normal Control; DM: Diabetes mellitus; DM + Gli-5: Diabetes mellitus + glibenclamide (5 mg/kg); DM + Salak-250: Diabetes mellitus + salak seed extract (250 mg/kg); DM + Salak-500: Diabetes mellitus + salak seed extract (500 mg/kg); DM + Salak-1000: Diabetes mellitus + salak seed extract (1000 mg/kg).

Figure 5A,B illustrate the fasting blood glucose (FBG) concentrations at various time intervals following the oral administration of glucose (0–120 min) in both normal and STZ-NA-induced diabetic mice, respectively. The AUC_0–120 min_ in normal mice treated with salak seed extract at doses of 500 and 1000 mg/kg was comparable to that in the normal control group (Figure 5C).

As shown in Figure 5B, treatment with both glibenclamide (5 mg/kg) and salak seed extract (500 and 1000 mg/kg) significantly decreased FBG levels at 30, 60, 90, and 120 min compared to the untreated diabetic group (*p* < 0.05). A comparison of the AUC_0–120 min_ (Figure 5D) found that oral administration of 500 and 1000 mg/kg of salak seed extract produced anti-hyperglycemic effects similar to glibenclamide. However, the FBS (0–120 min) and AUC_0–120 min_ after oral administration of 250 mg/kg of salak seed extract were not significantly different from those of the untreated DM mice.

#### 2.4.3. Anti-Hyperglycemic Effects of Salak Seed Extract on STZ-NA-Induced Diabetic Mice after Continuous Oral Administration for 28 Days

**Figure 6:**
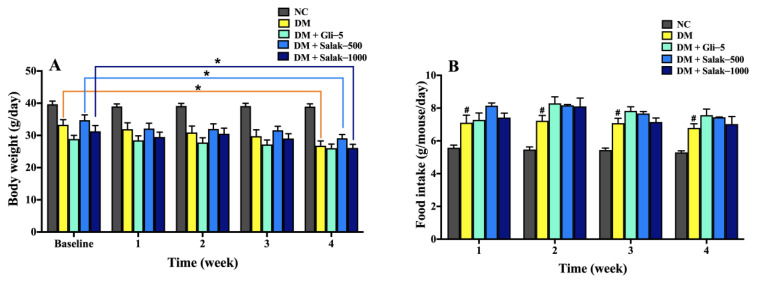
Effect of salak seed extract on body weight (**A**) and food intake (**B**). Data are expressed as mean ± SEM (n = 6–8/group). # *p* < 0.05 vs. NC, * *p* < 0.05 vs. DM. NC: Normal Control; DM: Diabetes mellitus; DM + Gli-5: Diabetes mellitus + glibenclamide (5 mg/kg); DM + Salak-500: Diabetes mellitus + salak seed extract (500 mg/kg); DM + Salak-1000: Diabetes mellitus + salak seed extract (1000 mg/kg).

After 28 days of continuous oral treatment with the salak seed extract, the body weight of DM mice decreased significantly, while their food intake increased compared to that of the normal control mice (Figure 6A,B). There was a significant decrease in the body weight in the diabetic groups compared with the normal control group (*p* < 0.05). In particular, the body weight of the untreated diabetic controls and the groups treated with 500 and 1000 mg/kg of the salak seed extract were significantly decreased in the last week of treatment compared to the baseline (*p* < 0.05). However, the mice in the normal control and glibenclamide-treated groups exhibited stable, unchanged body weights throughout the experiment. There was a significant increase in the food intake of diabetic mice as compared with that of the normal mice over the treatment period (*p* < 0.05) (Figure 6B). Food intake of the group treated with 5 mg/kg glibenclamide and that with 500 and 1000 mg/kg of salak seed extract remained unchanged compared with the untreated diabetic group.

**Figure 7:**
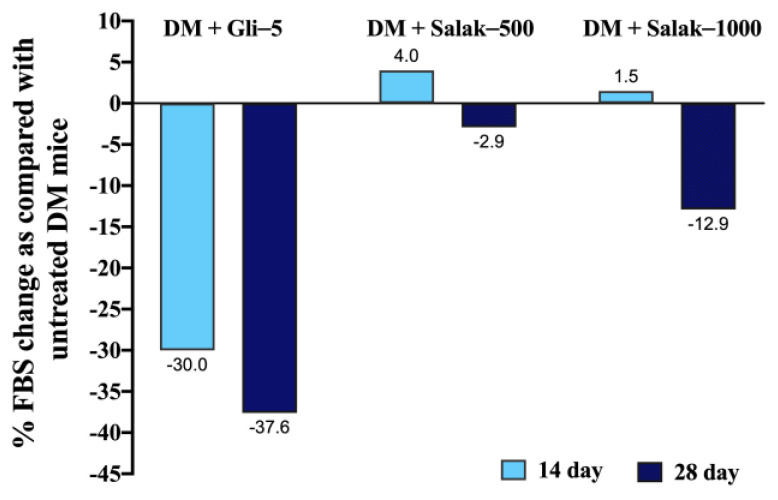
Effects of salak seed extract on the percentage change in fasting blood glucose as compared with untreated DM mice after oral administration for 28 days. Data are presented as % change compared with DM mice. DM: Diabetes mellitus; DM + Gli-5: Diabetes mellitus + glibenclamide (5 mg/kg); DM + Salak-500: Diabetes mellitus + salak seed extract (500 mg/kg); DM + Salak-1000: Diabetes mellitus + salak seed extract (1000 mg/kg).

As shown in Table 4, the fasting blood glucose levels in the untreated DM group were significantly higher than those in the normal control group (*p* < 0.05) and progressively raised until the end of the study. On the 14th and 28th days of treatment, the administration of 5 mg/kg glibenclamide significantly reduced the average FBG level of STZ-NA-induced diabetic mice with a percentage reduction of 30% and 37.6%, respectively compared to that in the untreated diabetic mice (*p* < 0.05). After the 14th day of treatment, the doses of 500 and 1000 mg/kg of salak seed extract did not show a significant effect on the FBG of STZ-NA-induced diabetic mice as compared with the untreated diabetic group. However, the FBG levels in DM mice treated with salak seed extract at doses of 500 and 1000 mg/kg for 28 days showed a trend towards being lower than those in the untreated DM mice, approximately 3% and 13%, respectively (Figure 7).

**Figure 8:**
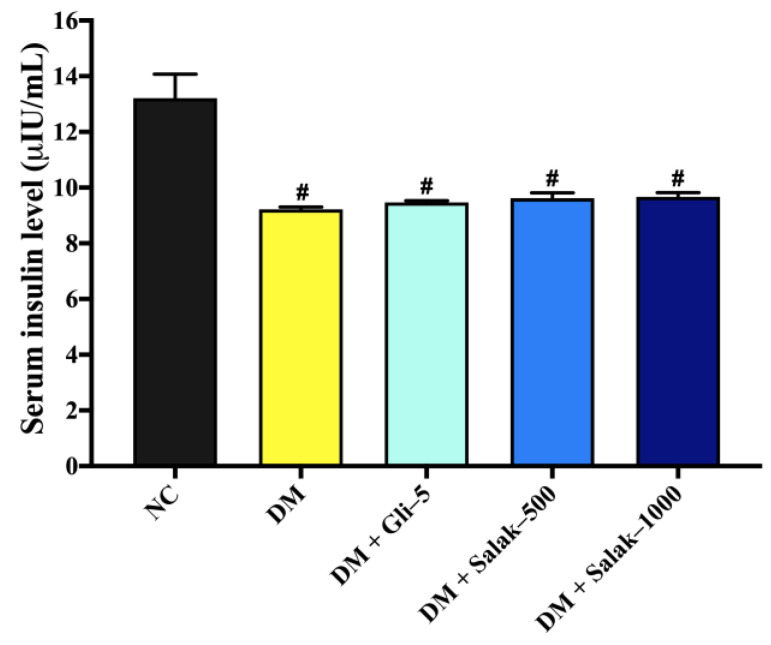
Effects of oral administration of salak seed extract for 28 days on serum insulin level. Data are expressed as mean ± SEM (n = 6–8). # *p* < 0.05 vs. NC. NC: Normal Control; DM: Diabetes mellitus; DM + Gli-5: Diabetes mellitus + glibenclamide (5 mg/kg); DM + Salak-500: Diabetes mellitus + salak seed extract (500 mg/kg); DM + Salak-1000: Diabetes mellitus + salak seed extract (1000 mg/kg).

Following the completion of the 28-day treatment regimen, the mice with diabetes mellitus exhibited a significant decrease in blood insulin levels in comparison to the control group of mice with normal physiological conditions (*p* < 0.05) (Figure 8). There were no statistically significant differences seen in the serum insulin levels between the groups treated with salak seed extract at doses of 500 and 1000 mg/kg, the group treated with glibenclamide, and the group of STZ-NA-induced diabetic mice that did not receive any treatment.

## 3. Discussion

Salak fruit and its seeds are currently processed as waste. However, previous research has shown that the seeds can be used to make salak seed coffee, also known as Salacca coffee, which is a functional beverage [13,14,15]. This coffee, produced from roasted salak seeds, has low caffeine content and is rich in antioxidants. Previous research has reported that salak seed extract contains several active phytochemical compounds, including antioxidants (436.91 mg/L), caffeine (0.207%), flavonoids (66.4 mg/100 g QE), and tannins (838.98 mg/100 TAE) [13]. In addition, powdered salak seed has demonstrated the capacity to absorb heavy metals such as cadmium, copper, and zinc, making it useful for cleaning industrial wastewater [14,16].

Saleh et al. [9] reported that caffeic acid, chlorogenic acid, levodopa, and ascorbic acid with some functional groups (C–H, C=C, C=O, and C–O) are responsible for α-glucosidase inhibition. Chlorogenic acid is present in large quantities in both salak fruit and salak seed [13,14]. It may have contributed to the inhibition of α-glucosidase activity and, thus, to the significant reduction in the plasma glucose levels found in our OGTT study, prompting us to investigate the anti-hyperglycemic effect of salak seeds.

This work represents the initial evidence of the hypoglycemic properties of salak seed extract in a murine model of diabetes mellitus caused by streptozotocin-nicotinamide, as assessed using an oral glucose tolerance test, where pretreatment with the extract before glucose loading markedly reduced FBG and AUC_0–120 min_ compared with untreated hyperglycemic mice. The anti-hyperglycemic effects of the seed extract were comparable to those of the antidiabetic drug, glibenclamide. Studies have shown that inhibition of α-glucosidase activity improves postprandial hyperglycemia [17,18]. α-Glucosidase inhibitors generally slow the liberation of glucose from oligosaccharides and disaccharides by interfering with enzymatic action in the brush border of the small intestine, leading to the reduction of postprandial plasma glucose levels. Therefore, reducing glucose production from carbohydrates through the inhibitions of α-glucosidase and intestinal glucose uptake can lower intestinal glucose absorption and improve glucose tolerance. A previous study has shown salak fruit extract to have an inhibitory effect on α-glucosidase activity [13]; however, the anti-hyperglycemic effects of salak seeds have not been mechanistically investigated. Consistent with the 60% ethanol-water salak fruit extract, which exhibited α-glucosidase inhibition with an IC_50_ value of 15.94 ± 2.52 μg/mL [13], salak seed extract exhibited strong, dose-dependent inhibitory effects on α-glucosidase activity, with an IC_50_ value of 16.28 ± 7.22 µg/mL. This was more potent than acarbose, suggesting a possible role of salak seed extract as a food supplement for lowering the glycemic index and reducing the risk of type 2 diabetes.

Using Lineweaver–Burk plots to determine the type of α-glucosidase inhibition, we found that salak seed extract exhibits non-competitive inhibition of the enzyme, with a K_i_ value of 24.81 μg/mL. Unlike acarbose, which exhibits competitive inhibition [19], salak seed extract contains several phytochemical compounds, including β-sitosteryl-3β-glucopyranoside-6’-O-fatty acid esters, triacylglycerols, linoleic acid, and chlorogenic acid. These may bind to different regions of the enzyme, rather than just the active site, causing concomitant apparent non-competitive inhibition, to differing degrees, in those regions.

In addition to the inhibition of α-glucosidase enzyme, the current approach for regulating postprandial blood glucose levels is the suppression of glucose absorption in the small intestine by the inhibition of sodium-dependent glucose transporter 1 (SGLT1) or glucose transporter 2 (GLUT2) [17]. Our study is the first to demonstrate that salak seed extract can inhibit glucose uptake in the well-characterized Caco-2 intestinal model. This could be among the mechanisms contributing to the hypoglycemic effects of salak seed extract in postprandial molecular hyperglycemic status. Consistent with these results, previous studies have demonstrated that chlorogenic acid has the ability to block the uptake of glucose in intestinal cells [20]. However, further molecular research is needed to evaluate how the salak seed extract interacts differently with the glucose transporters GLUT2 and SGLT1.

It is interesting to note that the salak seed extract did not exhibit a glucose-lowering effect on normoglycemic mice, despite its ability to delay glucose absorption in diabetic mice in the OGTT model. While this study does not provide a definitive explanation for this phenomenon, we speculate that the impact of salak seed extract on glucose metabolism remains within the bounds of normal homeostasis. However, a recent study identified functional and structural changes in type 2 DM mice, including an increase in the number of enterocytes in the villi of the small intestine [20]. This adaptation did not affect the transporters of SGLT1 and GLUT2 in the brush border. Consequently, the type 2 DM rats showed enhanced glucose absorption in the small intestine, which led to greater impairment of glucose tolerance than in normal rats [20]. Hence, it is plausible that the suppression of glucose absorption will exhibit a stronger effect in diabetic mice under hyperglycemic circumstances compared to normoglycemic animals. The lack of hypoglycemic activity under normoglycemic settings is a desirable characteristic of salak seed when considering its potential utilization in natural goods, dietary supplements, or food and beverage applications, such as salak seed coffee.

The involvement of oxidative stress in the etiology of diabetes suggests that antioxidant therapy has promise for its management. The antioxidant effects of salak seed extract have been verified in previous studies [13,21]. The present study found salak seed extract to markedly inhibit MDA production, the final product of lipid peroxidation, in a concentration-dependent manner. In accordance with previous research [21], salak seed extract exhibited antioxidant activity in both DPPH and ABTS assays. These antioxidant properties can be attributed to the presence of chlorogenic acid and other phenolic constituents. The antioxidant effect of salak seed extract in reducing MDA production during lipid peroxidation may be attributed to its hydrogen-donating capability, which is derived from its phenolic compounds. This prevents the generation of reactive oxygen species in the early stages of lipid peroxidation [22]. However, the effects of salak seed extract on endogenous prooxidants that contribute to oxidative stress in animal models of diabetes, such as superoxide dismutase (SOD) and glutathione peroxidase (GSHPx), should be examined in further studies to determine the potential antioxidant role of the seed extract in the prevention and/or treatment of diabetes and its complications.

Since 500 and 1000 mg/kg doses of the salak seed extract were shown to be effective for its anti-hyperglycemic actions in the present OGTT model, the same doses were subsequently employed in the 28-day continuous oral administration study. In contrast to the postprandial anti-hyperglycemic effects observed in the OGTT model, however, continuous oral administration of the extract did not produce a statistically significant antidiabetic effect during the period tested, albeit with an FBG-lowing tendency (Figure 7). A prior study discovered that chlorogenic acid, administered at a dose of 5 mg/kg along with tetrahydrocurcumin (80 mg/kg), exhibited antidiabetic effects in STZ-NA-induced DM rats after 45 days of treatment [23]. Pharmacokinetic investigations in rats have demonstrated that chlorogenic acid is quickly absorbed from the gastrointestinal tract, attaining maximum plasma concentrations within approximately 0.48 ± 0.29 h, and displaying a half-life of about 1.70 ± 0.24 h post oral administration [24]. The chlorogenic acid concentration in a 1000 mg/kg dose of the salak seed extract in this study was calculated to be around 5.5 mg/kg. Hence, we consider that the lack of antidiabetic effect in the present continuous administration case of salak seed extract may be attributed, at least partly, to the shorter period of daily administration (24 days vs. 45 days) and no usage of a concomitant antidiabetic agent (i.e., tetrahydrocurcumin co-administered in the previous study [23]). Taking the pharmacokinetic and pharmacodynamic variabilities of anti-hyperglycemic herbal polyphenols into consideration, synergistic combinations of salak seed extract together with other polyphenolic components could be explored to obtain more robust antidiabetic effects in the future.

In a previous study [21], rats were administered 70% ethanolic salak seed extract at doses of up to 1.12 g/kg for 7 consecutive days, revealing no signs of toxicity and no observable alterations in the histopathological structure of the kidneys, including the kidney glomeruli. However, it is important to note that an acute oral toxicity test had not been conducted in this prior study. Therefore, the current investigation followed the OECD toxicity test guideline 425 [25] and employed a limit test to assess the estimated LD_50_ value. After 14 days of oral administration of 2000 mg/kg of salak seed extract to 5 female mice, it was determined that the body weight of the mice remained unaffected when compared to the control group. Furthermore, the administration of salak seed extract at the dose of 2000 mg/kg did not result in any mortality during the entire duration of the study, and no significant behavioral changes were observed. These findings suggest that the estimated LD_50_ value of salak seed extract exceeds 2000 mg/kg, indicating a low level of toxicity.

Medicinal plants have been employed in managing type 2 diabetes due to their fewer or lack of side effects when compared to pharmaceuticals. Consumption of tea, coffee, or decaffeinated coffee has been shown to reduce the risk of type 2 DM [26]. This could also apply to salak seed coffee. The results of the present study confirm the potential use of salak seed extract as a functional food or supplement that could be used to lower postprandial hyperglycemia and exert antioxidant effects. This could be an effective treatment for prediabetes or a type 2 diabetes mellitus preventative. However, given the reported diuretic effect of salak seed extract, diuresis may be a potential side effect [21]. Future clinical studies are required to elucidate the pharmacology, toxicology, and pharmacokinetics of salak seed extract.

## 4. Materials and Methods

### 4.1. Identification of Plant Materials

Salak seeds from the Sala NoenWong cultivar were collected from a fruit garden in the Chanthaburi province of Thailand in May 2021 and authenticated at the Faculty of Pharmaceutical Sciences, Burapha University (sample specimen BV-210501).

### 4.2. Preparation of Salak Seed Extract

The plant’s seeds were rinsed with tap water, chopped into small pieces, and dried in a hot air oven at 55 °C for 24 h. The dried seeds were then pulverized into a powder using a grinder and passed through a 0.5 mm sieve. Extraction was conducted using a microwave-assisted extraction (MAE) technique with a solid-to-liquid ratio of 1:40. The solvent was 75% ethanol. The MAE (Utopia, Milan, Italy) technique utilizes a temperature-controlled system, which is set to reach 120 °C within 3 min and held at 120 °C for 10 min. The resulting sample was filtered through Whatman^®^ filter paper no. 1 (GE Healthcare, Waukesha, WI, USA), and the residue was extracted again in the same way. The pooled filtrates from each extraction were dried using a vacuum evaporator at 50 °C under reduced pressure, and the resulting extracts were stored in airtight containers at 4 °C. Only analytical-grade chemicals, solvents, and reagents were used in this study.

### 4.3. Quantification of Chlorogenic Acid by High-Performance Liquid Chromatography

Quantification of chlorogenic acid in salak seed extract was coinducted using a high-performance liquid chromatography (HPLC) method [27] on a Shimadzu (Shimadzu Corp., Tokyo, Japan) quaternary pump (LC-40D) equipped with a diode array detector (SPD-M40) set at 325 nm, a column oven (CT0-40C), and an autosampler (SIL-40C). Data were collected using Shimadzu LabSolutions software Version 5.87 SP1. A Luna^®^ C18 column (4.6 × 250 mm i.d., 5 µm) with a C18 guard column (Phenomenex, Torrance, CA, USA) was utilized. The detailed HPLC conditions used were the same with a previous study [27]. A calibration curve was produced by diluting standard chlorogenic acid with purity > 98.0% (Wuhan ChemFaces Biochemical Co., Ltd., Wuhan, China) within the range of 0.00625–0.1 mg/mL. Each sample was measured in triplicate.

### 4.4. Determination of Total Phenolic Content in Salak Seed Extract

The method of Sato et al. [28] was used to investigate the total phenolic content of the salak seed extract. The samples were evaluated in triplicate, and the results were presented as the mean ± standard deviation and expressed as mg equivalent of gallic acid per gram of dry weight (mg GAE/g DW).

### 4.5. Antioxidant Effects of Salak Seed Extract In Vitro

The antioxidant activity of salak seed extract was evaluated using DPPH, ABTS, and FRAP assays, as previously described [5]. Ascorbic acid was used as a positive control. Each determination was performed in triplicate, and inhibitory activity was expressed as the percentage of inhibition as follows [5]:

(1) $$\%\;inhibition=\frac{Ac-As}{A_{c}}\times 100$$

where A_c_ and A_s_ are the absorbances of the control and the sample, respectively.

Then, the IC50 values of DPPH and ABTS were calculated by plotting the percentage of inhibition against the log concentration of the salak seed extract.

For the FRAP assay, the extract’s ferric-reducing capacity was measured using the method described by Vongsak et al. [5]. FeSO_4_ was employed as the standard. The samples were evaluated in triplicate and the Fe^2+^ content was given as the mean ± SD of FeSO_4_ equivalent per gram of extract.

### 4.6. Effect of Salak Seed Extract on Malondialdehyde (MDA) Production In Vitro

Malondialdehyde is a major byproduct of lipid peroxidation during oxidative stress. MDA is often used as a marker of oxidative stress, which has been implicated in the pathophysiology of diabetes and its complications. [29,30]. The most applicable method to quantify lipid peroxidation status is the thiobarbituric acid reactive substances (TBARS) assay [30]. The amount of MDA produced was measured in vitro using a previous method [31] with slight modification. The percentage of inhibition was calculated using the following equation [30,31]:

(2) $$\%\;MDA\;production\;inhibitory\;activity=\left[\frac{A_{c}-A_{s}}{A_{c}}\right]\times 100$$

where A_c_ and A_s_ are the absorbances of the control and the sample, respectively.

The resulting MDA was quantified as MDA equivalent (mg MDA/g linoleic acid). The IC_50_ value was examined from the MDA production percentage inhibition activity versus the salak seed extract concentration.

### 4.7. In Vitro Hypoglycemic Effects of Salak Seed Extract

#### 4.7.1. Caco-2 Cell Culture and Cell Viability Assay

We conducted Caco-2 cell culture (ATCC^®^ HTB-37™) for in vitro anti-hyperglycemic evaluation, and subsequently an MTT assay to evaluate the cell viability under the exposure of salak seed extract, according to a previous method [32]. Briefly, salak seed extract (31.25–2000 μg/mL) was added to the monolayer Caco-2 cells and incubated for 4 h. The medium was replaced with MTT solution (5 mg/mL in PBS) and incubated for a further 4 h. The supernatant was removed and DMSO was added. The absorbance was measured at 550 nm using a microplate reader (Tecan, Männedorf, Switzerland). The percentage of cell viability was calculated using the following equation [32]:

(3) $$Cell\;viability\left(\%\right)=\frac{Absorbance\;of\;extract-Absorbance\;of\;treated\;cells}{Absorbance\;of\;control}\times 100$$

#### 4.7.2. Effect of Salak Seed Extract on Glucose Uptake in Caco-2 Cells

The cultured Caco-2 cells were incubated for 2 h with glucose-free Dulbecco’s modified Eagle medium (DMEM) and then for 30 min with Hanks’ balanced salt solution (HBSS) [33]. The cells were subjected to incubation for a duration of 10 min using HBSS solution that contained salak seed extract at concentrations ranging from 62.5 to 500 μg/mL. Additionally, a positive control was included in the form of phloridzin at a concentration of 10 μg/mL [34,35]. They were then replenished with glucose (0.55 mM) and incubated for 40 min. Then, ice-cold PBS was added. The cells were lysed with 1% Triton X-100 solution on ice, scraped, and centrifuged at 14,000 rpm for 15 min at 4 °C. The glucose content was quantified utilizing a commercially available glucose assay kit (Abcam, Cambridge, UK), whereas the protein content was assessed with Bradford’s technique. All treatments were performed in triplicate.

### 4.8. Effect of Salak Seed Extract on α-Glucosidase Activity In Vitro

The inhibitory effect of the salak seed extract on α-glucosidase activity was determined using a spectrophotometric method, according to a previous study [34]. The percentage of inhibition was calculated using Equation (4) [34]:

(4) $$\alpha -Glucosidase\;inhibitory\;activity\left(\%\right)=\left[\frac{A_{c}-A_{s}}{A_{c}}\right]\times 100$$

where A_c_ and A_s_ are the absorbances of the control and the sample, respectively.

The IC_50_ value was determined by the construction of a graph that illustrates the relationship between the percentage of α-glucosidase activity inhibition and the logarithmic concentration of the salak seed extract. In order to ascertain the specific enzymes that are hindered by the salak seed extract, resulting in the decrease in α-glucosidase activity, a Lineweaver–Burk plot analysis was conducted. The Michaelis–Menten constant (K_m_), the inhibition constant (K_i_), and the maximum metabolic rate (V_max_)were assessed by non-linear regression analysis utilizing the Solver add-in in Microsoft Excel 2010, based on the provided equation [34]:

(5) $$\nu =\frac{V_{max}\times S}{K_{m}+S\;\left[1+\frac{I}{K_{i}}\right]}$$

where ν represents the reaction velocity (μM/min) and S and I represent the substrate and inhibitor concentrations in μM and μg/mL, respectively.

### 4.9. In Vivo Antihyperglycemic Activity of Salak Seed Extract in STZ-NA-Induced DM Mice

#### 4.9.1. Animals

Male ICR mice (30–40 g) were housed in the Animal Center at the Faculty of Pharmacy, Mahidol University at a constant temperature of 25 °C and with a 12-h light-dark cycle. They were given ad libitum access to standard food and water, except when fasting prior to glucose experiments. The experimental protocol was approved by the Animal Ethics Committee of the Faculty of Pharmacy, Mahidol University (permission number: PYR003/2022).

#### 4.9.2. Oral Acute Toxicity Test

The acute toxicity assay of the salak seed extract followed the guidelines of the Organization for Economic Cooperation and Development (OECD), specifically test guidelines 425 (up and down procedure), in accordance with the principles of the limit test [25]. The limit test, utilizing a maximum of 5 animals, was conducted at a dosage of 2000 mg/kg. Five nulliparous, non-pregnant female mice weighing between 30–35 g were employed for this experiment. Following a 7-day adaptation period, the mice underwent a 3–4 h fasting period prior to dosing, while having unrestricted access to water. The first group of mice received a single oral dose of 2000 mg/kg of salak seed extract, dissolved in water at a volume of 1 mL/100 g via oral gavage. Surviving mice were closely monitored within the first 30 min to detect any toxic reactions. If the first mouse survived without showing signs of toxicity, an additional 4 mice were sequentially dosed with the same amount under identical conditions, resulting in a total of 5 mice in the treatment group. The same procedure was applied to a control group of 5 mice, which were treated with water in the same volume as the salak-treated group. Food was provided 1–2 h after dosing. Both groups were closely observed for any signs of toxicity i.e., changes in the fur and skin color, eyes, respiration, convulsion, and diarrhea, within the first 4 h and at regular intervals for a total of 14 days. Daily monitoring and recording of weights and symptoms of toxicity were conducted. At the conclusion of the study, the mice were weighed and euthanized using CO_2_ inhalation. If three or more animals died during the experiment, the limit test was terminated, and the main test was initiated. The LD_50_ value was considered to be higher than the test dose (2000 mg/kg) if three or more animals survived.

#### 4.9.3. Induction of Type 2 Diabetes in Mice

After a single intraperitoneal (i.p.) dose of 120 mg/kg nicotinamide (NA) dissolved in saline, overnight-fasted mice (12 h) were injected i.p. with 180 mg/kg of STZ dissolved in 0.1 M of citrate buffer (pH 4.5). The assessment of diabetes development was conducted after a period of 14–21 days, during which blood glucose levels were measured following an overnight fasting period. Blood samples were obtained from the tail vein, and glucose concentrations were assessed using a glucometer equipped with glucose test strips manufactured by Roche Diabetes Care, Inc., Indianapolis, IN, USA. In our investigation, mice were chosen as subjects if they had fasting blood glucose (FBG) levels beyond 200 mg/dL, indicating the presence of diabetes mellitus (DM) [34].

#### 4.9.4. Oral Glucose Tolerance Testing (OGTT) in Normal and Diabetic Mice

The anti-hyperglycemic effects of salak seed extract on both normal and STZ-NA-induced DM mice were evaluated by oral glucose tolerance tests (OGTT). A direct toxicity study of salak seed extract has not previously been conducted. However, a previous in vivo study using Wistar rats showed that there were no noticeable signs of toxicity, and no mortality was observed after daily oral administration of 70% ethanol salak seed extract at doses of 0.28 g, 0.56 g, and 1.12 g/kg for 7 days [22]. Furthermore, salak seed extract has been commercially available as coffee and a beverage in certain countries, such as Indonesia, which confirms the safety of the extract [13]. As a result, favorable doses (250, 500, and 1000 mg/kg) of the salak seed extract were selected for this study.

The methods of Karthikesan et al. [23] and Sato et al. [28] were employed in this study with some modifications. In order to investigate the potential hypoglycemic effects of the salak seed extract in normal mice, the extract was diluted in water and orally supplied to mice that had fasted for 12 h. This administration took place 30 min before the oral glucose loading, which consisted of a dosage of 2 g/kg. In a separate experiment, mice with type 2 diabetes produced by overnight fasting and streptozotocin-nicotinamide administration were separated into five distinct groups. The groups of diabetic rats induced by STZ-NA were given oral administrations of water, glibenclamide (5 mg/kg) as a positive control, or salak seed extract (250, 500, and 1000 mg/kg) 30 min prior to the oral administration of a glucose solution (2 g/kg). Blood samples were collected from the tail vein at several time points: baseline (0), 30, 60, 90, and 120 min. These samples were then subjected to blood glucose analysis using a glucometer equipped with glucose test strips manufactured by Roche Diabetes Care, Inc., located in the United States. The calculation of the area under the curve (AUC) for the concentrations of FBG throughout a time period of 0–120 min was performed using the linear trapezoidal rule approach. [34]:

(6) $${AUC}_{0-120\;min}=\frac{\left[C_{0}+C_{30}\right]\cdot \left[t_{30}{-t}_{0}\right]}{2}+\frac{\left[C_{30}+C_{60}\right]\cdot \left[t_{60}-t_{30}\right]}{2}+\frac{\left[C_{60}+C_{90}\right]\cdot \left[t_{90}-t_{60}\right]}{2}+\frac{\left[C_{90}+C_{120}\right]\cdot \left[t_{120}-t_{90}\right]}{2}$$

where C_0_, C_30_, C_60_, C_90_, and C_120_ are the FBG concentrations (mg/dL) 0, 30, 60, 90, and 120 min after glucose administration, respectively; t_0_, t_30_, t_60_, t_90_, and t_120_ are the times (min) after glucose administration.

#### 4.9.5. Anti-Hyperglycemic Effects of Salak Seed Extract on STZ-NA-Induced DM Mice after 28-Day Continuous Daily Administration

The determination of anti-hyperglycemic effects was conducted as described previously [34]. We selected 500 mg/kg and 1000 mg/kg doses of the salak seed extract for 28-day continuous oral daily administration. The mice were divided into 5 groups (n = 6–8) as follows:

1. Normal Control group (NC): Mice in this group received water (serving as the normal control group).
2. Diabetes Mellitus group (DM): Mice with diabetes administered with water.
3. DM + Gli-5: Mice with diabetes administered with 5 mg/kg of glibenclamide (serving as a positive control group).
4. DM + Salak-500: Mice with diabetes administered with 500 mg/kg of salak seed extract.
5. DM + Salak-1000: Mice with diabetes administered with 1000 mg/kg of salak seed extract.

All treatments were conducted once daily via oral gavage for 28 days. During the 28-day treatment period, the body weight and average food intake of the mice were measured daily. The measurement of fasting blood glucose levels was conducted following a 12 h fasting period on days 0, 14, and 28 of the treatment periods. At the end of the trial, the mice passed away by an overdose of CO_2_ inhalation. Blood was collected via cardiac puncture and then centrifuged at 5000 rpm, 4 °C for 8 min to obtain serum and used in the next studies. Serum insulin levels were measured using a mouse insulin ELISA kit (Fujifilm, Tokyo, Japan), according to the manufacturer’s guidelines.

### 4.10. Statistical Analysis

The data from in vitro studies were presented as the mean ± standard deviation (SD), while the data from in vivo studies were presented as mean ± standard error of mean (SEM). The significant differences were analyzed using one-way ANOVA, followed by post-hoc Tukey’s multiple comparisons. Statistical testing was conducted using SPSS version 18 (SPSS Inc., Chicago, IL, USA). The statistical significance level was set at *p* < 0.05.

## 5. Conclusions

This study demonstrated the salak seed extract’s capability to mitigate postprandial hyperglycemia in STZ-NA-induced diabetic mice. This in vivo finding was rationalized by in vitro experiments which clarified its effects on glucose-maintaining molecular mechanisms, i.e., α-glucosidase activity and intestinal glucose uptake. Furthermore, our investigation has revealed the antioxidant effects of salak seed extract, notably the inhibition of MDA production as a consequential outcome of lipid peroxidation. The salak seed extract is considered safe, as its LD_50_ value exceeds 2000 mg/kg. These findings suggest the potential of salak seeds as a valuable component for functional health products and beverages tailored to support the management of postprandial hyperglycemia, which may further contribute to various complications associated with diabetes, such as cardiovascular diseases. We anticipate further research based on our findings, which could help develop additional applications and integration of salak seeds into natural antidiabetic therapies.

## Figures and Tables

**Table 1:** Total phenolic content, FRAP value, and IC_50_ values for DPPH and ABTS assays in the salak seed extract and ascorbic acid (positive control).

	Total Phenolic Content [mg GAE/g Extract]	FRAP Value [mg Fe^2+^/g Extract]	DPPH Assay IC_50_ [µg/mL]	ABTS Assay IC_50_ [µg/mL]
Salak seed extract	15.60 ± 0.79	17.92 ± 0.10	126.17 ± 1.76	380.35 ± 12.56
Ascorbic acid	N/A	94.71 ± 2.62	5.33 ± 0.1	11.50 ± 0.45

Data are expressed as mean ± SD (n = 3). GAE: gallic acid equivalent; IC_50_: 50% inhibitory concentration; FRAP: ferric-reducing antioxidant power; DPPH: 2,2-diphenyl-1-picrylhydrazyl; ABTS: 2,2-azino-bis (3-ethylbenzothiazoline-6-sulfonate; N/A: not applied for ascorbic acid.

**Table 2:** Toxicity symptoms in control and salak-treated groups in the acute toxicity test.

Symptoms of Toxicity	Baseline		1-Day		7-Day		14-Day	
	Control	Salak	Control	Salak	Control	Salak	Control	Salak
Fur and skin color	N	N	N	N	N	N	N	N
Eyes	N	N	N	N	N	N	N	N
Respiration	N	N	N	N	N	N	N	N
Convulsion	NF	NF	NF	NF	NF	NF	NF	NF
Diarrhea	NF	NF	NF	NF	NF	NF	NF	NF

N = Normal; NF = Not Found.

**Table 3:** Effects of the salak seed extract on mortality and body weight of mice in acute toxicity study.

Treatment	Mortality (Dead/Treated Mice)	Body Weight (g)		
		Baseline	7-Day	14-Day
Water (control)	0/5	31.04 ± 0.92	32.23 ± 0.17	33.50 ± 0.67
2000 mg/kg salak seed extract	0/5	31.26 ± 0.64	33.63 ± 0.35	33.63 ± 0.21

Data are expressed as mean ± SEM (n = 5).

**Table 4:** Effects of 28-day oral administration of salak seed extract on FBG in STZ-NA-induced DM mice.

Groups	Fasting Blood Glucose (mg/dL)		
	0 Day	14 Days	28 Days
NC	81.83 ± 6.59	94.50 ± 4.13	93.67 ± 6.18
DM	315.38 ± 35.61 ^#^	295.20 ± 35.07 ^#^	436.43 ± 35.58 ^#^
DM + Gli-5	318.29 ± 35.08	205.40 ± 62.95 *	272.20 ± 47.38 *
DM + Salak-500	326.00 ± 32.94	307.00 ± 41.89	423.80 ± 50.49
DM + Salak-1000	312.25 ± 31.43	299.40 ± 75.21	386.60 ± 34.59

Data are expressed as mean ± SEM (n = 6–8). # *p* < 0.05 vs. NC, * *p* < 0.05 vs. DM. NC: Normal Control; DM: Diabetes mellitus; DM + Gli-5: Diabetes mellitus + glibenclamide (5 mg/kg); DM + Salak-500: Diabetes mellitus + salak seed extract (500 mg/kg); DM + Salak-1000: Diabetes mellitus + salak seed extract (1000 mg/kg).

## Data Availability

The data presented in this study are available on request from the corresponding author.
